# Evaluation of the Fluorescence Polarization Assay as a Rapid On-Spot Test for Ruminant Brucellosis in Côte d'Ivoire

**DOI:** 10.3389/fvets.2019.00287

**Published:** 2019-09-10

**Authors:** Laura C. Falzon, Sylvain Traoré, Vessaly Kallo, Jean-Baptiste Assamoi, Bassirou Bonfoh, Esther Schelling

**Affiliations:** ^1^Institute of Infection and Global Health, University of Liverpool, Liverpool, United Kingdom; ^2^International Livestock Research Institute, Nairobi, Kenya; ^3^Veterinary Public Health Institute, University of Bern, Bern, Switzerland; ^4^Université Pelefero Gon Coulibaly de Korhogo, Korhogo, Côte d'Ivoire; ^5^Centre Suisse de Recherches Scientifiques en Côte d'Ivoire, Abidjan, Côte d'Ivoire; ^6^Direction des Services Vétérinaires de Côte d'Ivoire, Abidjan, Côte d'Ivoire; ^7^Swiss Tropical and Public Health Institute, Basel, Switzerland

**Keywords:** Brucella, Rose Bengal Test, Fluorescence Polarization Assay, Kappa agreement, McNemar's Chi-squared test, cows, sheep, goats

## Abstract

Brucellosis is a zoonosis of economic and public health concern. While most diagnostic tests for brucellosis can only be performed in the laboratory, the Fluorescence Polarization Assay (FPA) was developed as a rapid point-of-care field test. This pilot project aimed to validate the use of FPA for rapid diagnosis of ruminant brucellosis on the field, and to compare the FPA performance with that of the more commonly used Rose Bengal Test (RBT). Blood samples were first collected from ruminants in a livestock market, and later from a nearby slaughterhouse in Port Bouët, Abidjan, Côte d'Ivoire. Samples collected in the livestock market were processed and tested with the FPA in a central laboratory, while samples collected in the slaughterhouse were processed immediately and the FPA was performed on site. To assess the FPA intra-test agreement, a portion of the serum samples tested at the slaughterhouse were re-tested with the FPA in the laboratory later the same day. To assess inter-test agreement, all serum samples were retested with the RBT. A total of 232 samples were tested with the FPA, 106 and 126 from the livestock market and slaughterhouse, respectively. Of these, 26 tested positive and 39 were doubtful for brucellosis. The FPA was repeated on 28 of the samples collected at the slaughterhouse, and comparison of results indicated a moderate intra-test agreement (Kappa = 0.41). The agreement improved when the doubtful category was treated as negative (Kappa = 0.65), and when cattle were excluded (Kappa = 0.56 to 0.61). The RBT was performed on 229 samples, and of these 10 tested positive. A comparison of FPA and RBT results indicated poor agreement (Kappa = 0.00); this improved to slight when only samples taken at the livestock market and tested in the laboratory were considered (Kappa = 0.14). The FPA did not perform well in tropical field conditions, possibly due to the high ambient temperatures in the slaughterhouse. Moreover, a difference in performance was noted in relation to the species tested, whereby the FPA seemed to perform better on sheep and goat samples, compared to cattle samples. These findings highlight that further adjustments are needed before implementing the FPA on the field.

## Introduction

Brucellosis is one of the most economically important zoonoses worldwide, with both public health and international trade implications (1–3). It is caused by a Gram-negative bacterium, of which twelve species have been described to date (4). Of these, the most important from a public health perspective are *Brucella melitensis* and *Brucella abortus*, which commonly infect small ruminants and cattle, respectively (5, 6). These zoonotic pathogens can be transmitted to humans through consumption of unpasteurized dairy products or handling of infected biological material (7, 8).

Brucellosis was first described in Malta in the 1850s. The causative agent, initially called *Micrococcus melitensis*, was isolated in 1887, while Sir Temistoceles Zammit recognized the zoonotic nature of this disease and the pivotal role of goats as reservoirs in 1905 (9, 10). Since then, brucellosis has been controlled or eliminated in countries such as Australia, the USA, and some European countries (1, 11). However, the disease persists endemically in several other areas, such as the Mediterranean basin, Middle East, Central and South America, Asia and Africa (1, 2, 6, 7).

One of the main hurdles in managing and controlling brucellosis is the lack of a perfect reference test. Case definition of the disease is done through isolation and identification of the bacterial agent (5, 12). However, bacterial culture is time-consuming and requires special media and a Biosafety level 3 laboratory, limiting its widespread use (3, 11, 13). Therefore, serological tests are more commonly used. These include agglutination tests, such as the Rose Bengal Test (RBT), Complement Fixation Tests, immunosorbent assays, and primary binding tests, such as ELISA and the Fluorescence Polarization Assay (FPA) (5, 12).

The FPA relies on the fact that molecules in a solution rotate randomly, resulting in the depolarization of plane polarized light (14). As this movement is inversely proportional to the molecular weight, fluorescein-bound antigens that react with antibodies have a reduced movement, with a consequent reduction in light depolarization. This change in the rate of depolarization can be measured by the FPA in milliPolarization units, thus providing a rapid and objective test result (15). Validation studies have shown FPA to be highly accurate, with reported sensitivities of 99.3, 94.9, and 91.5%, and specificities of 100, 99.4, and 98.6% in cattle, goats, and sheep, respectively (15). Furthermore, the FPA is described as a rugged test appropriate for use in the field (12, 15). However, there is scant information on the performance of the FPA as a rapid on-spot test for diagnosis of ruminant brucellosis in low-resource settings and tropic conditions.

The objectives of this project were therefore 2-fold: (i) to evaluate the effectiveness and feasibility of the FPA as a rapid on-spot test in a slaughterhouse in Abidjan, Côte d'Ivoire, and (ii) to compare its performance with the RBT. The rationale for this study was to determine whether the FPA could be used to screen and identify positive animals before slaughter, so that their organs could then be collected for further testing after. These findings were to inform the design of a larger epidemiological study evaluating the prevalence and parameters of diagnostic tests for brucellosis planned for this study area.

## Methods

### Study Area

This project was conducted between April and May 2015 in a livestock market and slaughterhouse in Port Bouët, Abidjan, in Côte d'Ivoire. The study site selection was based on the fact that a larger epidemiological study was planned for this study area. The ruminant slaughterhouse was open all day, every day, and slaughtered between 200 and 300 animals daily (16). The livestock market was located close to the slaughterhouse, and animals owned by different traders were kept in separate pens.

### Animal Sampling

Animal selection was done purposively based on the owners' willingness to have their animals sampled, and other logistical factors, such as ease of restraint.

The livestock market was visited twice. During each visit, animal owners were approached and the study rationale explained. If the owners accepted, the animals were manually restrained and 6 ml of blood were collected from the jugular vein into blood tubes using a Vacutainer cuff with disposable needles.

The slaughterhouse was visited four times. Many owners were not keen to have their animals sampled prior to slaughter as they feared that this might compromise the meat quality later. Blood was therefore collected by filling in the vacutainers with the first blood drawn at slaughter.

### Sample Processing and Testing for Brucellosis

#### Fluorescence Polarization Assay

**Figure 1:**
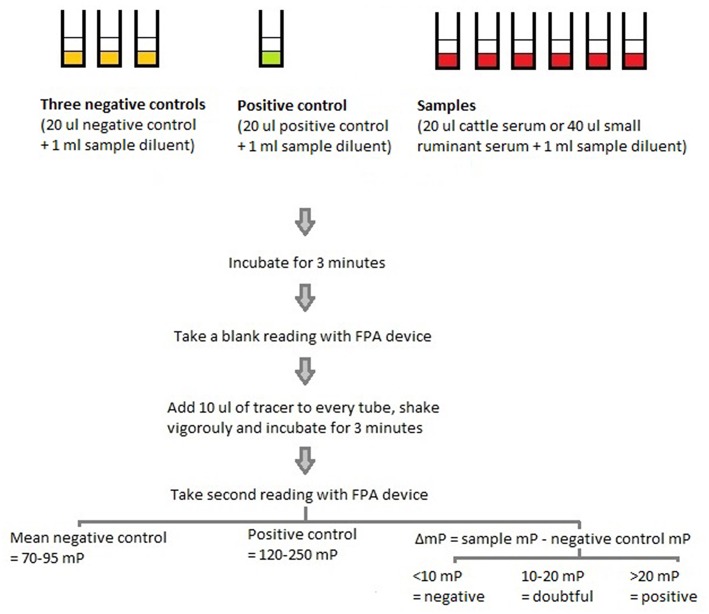
The protocol used to test serum samples obtained from cattle and small ruminants in a livestock market and slaughterhouse in Abidjan, Côte d'Ivoire, with the Fluorescence Polarization Assay.

Blood samples collected in the livestock market were stored on ice in a cool box and transported to the laboratory at the Center Suisse de Recherches Scientifique en Côte d'Ivoire (CSRS) in Abidjan, where they were processed following the protocol described in Figure 1, as per the manufacturers' instructions. Specifically, all samples and reagents were first allowed to reach the same room temperature of 22°C. Then, 20 μl of cattle serum or 40 μl of sheep or goat serum were mixed with 1 ml of sample diluent and left to stand for 3 min. Concurrently, 3 negative controls (20 μl of negative control + 1 ml of sample diluent), and 1 positive control (20 μl of positive control + 1 ml of sample diluent) were prepared. After three min, a first reading (also referred to as “blank reading”) of all samples and controls was taken using the FPA device (Brucella FPA^®^, Diachemix, LLC, USA), and the milliPolarization units were recorded. Then, 10 μl of the tracer (O-polysaccharide extracted from *B. abortus* bacteria and conjugated with fluorescein) were added to each tube; the tubes were shaken vigorously and left to stand for another 3 min, after which a second reading was taken.

The negative controls were to have a reading of 70–95 mP, while the positive controls were to have a reading of 120–250 mP. For the samples, the change in mP was determined by subtracting the negative control mP (based on the mean of the three negative controls) from each sample mP (i.e., ΔmP = sample mP—mean negative control mP). The ΔmP was then used to determine the status of the animal. A ΔmP <10, between 10 and 20, or >20 was considered indicative of a negative, doubtful, or positive brucellosis status, respectively.

**Figure 2:**
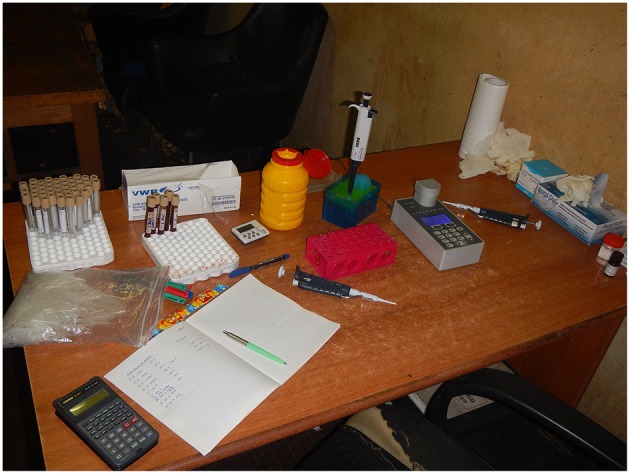
Field set-up to perform the Fluorescence Polarization Assay (FPA) in a slaughterhouse in Abidjan, Côte d'Ivoire.

Blood samples collected in the slaughterhouse were taken to a small room identified where the FPA device was set up (Figure 2). The blood samples were left to rest for 5–15 min to allow the blood to separate, after which they were processed and tested following the same protocol described above. Additionally, samples collected on the last slaughterhouse visit were tested twice to assess the intra-test agreement: once at the slaughterhouse shortly after collection, and again at the laboratory 6–8 h later. All FPA tests were carried out by the main author.

#### Rose Bengal Test

All serum samples were stored in Eppendorf tubes and kept at −20°C until further testing. The serum samples were then thawed and tested for brucellosis using the RBT, ensuring a constant ambient temperature of 27°C through air-conditioning of the laboratory where the test was performed. In cattle, a proportion of 30 μl of serum and 30 μl of antigen (*B. abortus* strain S 99, Bio-Rad^ND^) were mixed on a plate (17), while in sheep and goats the RBT was performed by mixing 75 μl of serum and 25 μl of antigen (18). The plate was rocked gently clockwise and counter-clockwise for exactly 4 min. A sample was considered positive if any perceptible agglutination reaction occurred within those 4 min. Samples were considered negative if no reaction was observed after 4 min. Single blinding was performed, whereby those performing the RBT were not aware of the FPA results so as to avoid misclassification bias.

### Data Cleaning and Analysis

All data were entered manually into a Microsoft Excel spreadsheet, and data cleaning and analysis were carried out using Stata Statistical Software: Release 14 (College Station, TX: StataCorp LP).

The number of animals that tested doubtful or positive for brucellosis was determined. This was then used to determine the intra-test Kappa agreement for FPA results obtained in the slaughterhouse and in the laboratory, and the inter-test Kappa agreement between FPA and RBT results. Since the FPA results were on a multi-level scale (i.e., negative, doubtful, or positive), while the RBT results were dichotomous (i.e., negative or positive), multiple Kappa agreements were computed. Specifically, a weighted Kappa was first computed to compare the agreement between FPA results obtained in the slaughterhouse and in the laboratory. This was followed by Kappa agreements where the doubtful category was considered as either negative or positive. Similarly, for the Kappa agreement between FPA and RBT tests, the doubtful category was considered as either negative or positive. Lastly, Kappa agreements were determined for samples from all animals, and for samples from sheep and goats only, and for samples from cows only. For each Kappa computation, a McNemar's Chi^2^ test was computed to determine whether the contingency tables for the two compared tests were symmetrically distributed.

The Kappa agreement scores were interpreted using the scale described by Dohoo et al. (19), where values ≤ 0.0, or between 0.01–0.20, 0.21–0.40, 0.41–0.60, 0.61–0.80, and 0.81–1.00, were considered indicative of poor, slight, fair, moderate, substantial and almost perfect agreement, respectively. For the McNemar's Chi^2^ test, a *p*-value <0.05 was considered statistically significant, indicative that the contingency tables were asymmetrically distributed.

## Ethical Approval

Approval was obtained from the National Ethics Committee of the “Ministère de la Santé et de la Lutte contre le Sida” of Côte d'Ivoire (N°71/MSLS/CNER-dkn) and the “Direction Générale de Recherche Scientifique et de l'Innovation Technologique du Ministère de l'Enseignement Supérieur et de la Recherche Scientifique” (N° 089/ MESRS/DGRSIT/KYS/tm). Authorisation was also obtained from the district and the Directorate of Veterinary Services following a letter from the CSRS. Consent to sample was obtained from the animal owner, no animal owner information was collected, and all animal samples and results were coded and treated confidentially.

## Results

### Fluorescence Polarization Assay Results

A total of 236 ruminants were sampled: 106 and 130 from the livestock market and slaughterhouse, respectively.

In the livestock market, 101 sheep and 5 goats were sampled, and all samples were tested in the CSRS laboratory on the same day of collection. Of the 101 sheep tested, 8 were positive and 23 were considered doubtful for brucellosis, while of the 5 goats tested, 3 were positive and 1 was considered doubtful for brucellosis (Table 1).

**Table 1:** Number of ruminant samples that were tested with the Fluorescence Polarization Assay (FPA), and number of samples that tested doubtful or positive for brucellosis, in a slaughterhouse and livestock market in Port Bouët, Abidjan, Côte d'Ivoire.

	**No. Cows Sampled**	**No. Cows Doubtful**	**No. Cows Positive**	**No. Sheep Sampled**	**No. Sheep Doubtful**	**No. Sheep Positive**	**No. Goats Sampled**	**No. Goats Doubtful**	**No. Goats Positive**
**SLAUGHTERHOUSE**									
1st visit	21	2	1	9	2	1	3	0	0
2nd visit	10	0	1	19	1	3	21	1	2
3rd visit	3	0	1	8	5	0	4	2	0
4th visit^*^	10	0	1	13	2	4	5	0	1
**Slaughterhouse total**	**44**	**2**	**4**	**49**	**10**	**8**	**33**	**3**	**3**
**LIVESTOCK MARKET**									
1st visit	0			44	8	3	5	1	3
2nd visit	0			57	15	5	0		
**Livestock market total**				**101**	**23**	**8**	**5**	**1**	**3**
**Overall total**	**44**	**2**	**4**	**150**	**33**	**16**	**38**	**4**	**6**

* *Results shown here are based on the first testing done in the slaughterhouse*.

The 130 animals sampled in the slaughterhouse comprised 45 cows, 51 sheep, and 34 goats. However, only 126 of these were tested with the FPA as 4 of the blood samples were insufficient to run the test. Of these 126 samples, 15 tested positive for brucellosis while another 15 were doubtful (Table 1).

#### Intra-Test Agreement Between FPA Results Obtained in the Slaughterhouse and Laboratory

On the fourth day of slaughterhouse sampling, the 28 samples collected were tested twice: once at the slaughterhouse shortly after collection, and then again in the laboratory 6–8 h later. The intra-test Kappa for all samples was moderate (0.41), and it improved when the doubtful category was considered as negative (0.50), compared to when the doubtful category was considered as positive (Kappa = 0.34; Table 2). The Kappa agreement improved when it was computed for sheep and goat samples only, ranging between 0.56 when the doubtful category was considered as negative, and 0.61 when the doubtful category was considered as positive. On the other hand, the Kappa agreement was poor when only cow samples were considered, regardless of the scenario under consideration (Kappa = −0.19 to −0.11). The McNemar's Chi^2^ test was not statistically significant, indicating that the contingency tables for the two compared results were symmetrically distributed in all the considered scenarios.

**Table 2:** Kappa agreement between FPA results obtained for the 28 samples collected during the fourth slaughterhouse visit, and tested first at the slaughterhouse and then in the Centre Suisse de Recherches Scientifiques en Côte d'Ivoire laboratory in Abidjan, Côte d'Ivoire.

	**All samples (*n* = 28)**	**Only sheep and goat samples (*n* = 18)**	**Only cows (*n* = 10)**
**Treating doubtful samples as doubtful (i.e., multi-level scale)**			
Weighted Kappa McNemar's Chi^2^ test	0.41 [moderate] *p*-value = 0.10	0.58 [moderate] *p* –value = 0.32	−0.17 [poor] *p*-value = 0.18
**Treating doubtful samples as negative**			
Kappa McNemar's Chi^2^ Test	0.50 [moderate] *p*-value = 0.65	0.61 [substantial] *p*-value = 0.56	−0.11 [poor] *p*-value = 1.00
**Treating doubtful samples as positive**			
Kappa McNemar's Chi^2^ Test	0.34 [fair] *p*-value = 0.10	0.56 [moderate] *p*-value = 0.32	−0.19 [poor] *p*-value = 0.18

### Rose Bengal Test Results

A total of 229 serum samples were tested with the RBT: 104 samples from the livestock market and 125 samples from the slaughterhouse. Of the 104 samples collected at the livestock market, 2 sheep samples tested positive, while of the 125 samples collected at the slaughterhouse, 8 tested positive (4 goats, 2 cows, and 2 sheep).

#### Inter-test Agreement Between FPA and RBT

The inter-test agreement ranged from poor to slight (Kappa = −0.12 to 0.20); it improved when only cow samples were considered and the FPA doubtful category was considered as positive (Kappa = 0.20; Table 3). Moreover, the Kappa agreement between the FPA results obtained in the laboratory and the RBT was slightly better (Kappa = 0.02–0.14), compared to the overall agreement (i.e., samples collected in both livestock market and slaughterhouse), or that between FPA results obtained in the slaughterhouse and the RBT results.

**Table 3:** Kappa agreement between FPA and Rose Bengal Test (RBT) results for 229 ruminant serum samples collected from a livestock market and slaughterhouse in Abidjan, Côte d'Ivoire.

**Both slaughterhouse and livestock samples**			
	**All samples (*****n*** **=** **229)**	**Only sheep and goats (*****n*** **=** **184)**	**Only cows (*****n*** **=** **45)**
**Treating doubtful samples as negative**			
Kappa McNemar's Chi^2^ Test	0.00 [poor] *p*-value <0.01^*^	0.01 [slight] *p*-value = 0.01^*^	−0.06 [poor] *p*-value = 0.41
**Treating doubtful samples as positive**			
Kappa McNemar's Chi^2^ Test	−0.02 [poor] *p*-value <0.01^*^	−0.04 [poor] *p*-value <0.01^*^	0.20 [slight] *p*-value = 0.10
**Only slaughterhouse samples**			
	**All samples(*****n*** **=** **125)**	**Only sheep and goats (*****n*** **=** **80)**	**Only cows (*****n*** **=** **45)**
**Treating doubtful samples as negative**			
Kappa McNemar's Chi^2^ Test	−0.09 [poor] *p*-value = 0.09	−0.10 [poor] *p*-value = 0.13	−0.06 [poor] *p*-value = 0.41
**Treating doubtful samples as positive**			
Kappa McNemar's Chi^2^ Test	−0.04 [poor] *p*-value <0.01^*^	−0.12 [poor] *p*-value <0.01^*^	0.20 [slight] *p*-value = 0.10
**Only livestock market samples**			
	**All samples (*****n*** **=** 104)^**^		
**Treating doubtful samples as negative**			
Kappa McNemar's Chi^2^ Test	0.14 [slight] *p*-value = 0.01^*^		
**Treating doubtful samples as positive**			
Kappa McNemar's Chi^2^ Test	0.02 [slight] *p*-value <0.01^*^		

* *Statistically significant p-value*.

** *No cows were sampled in the livestock market*.

The McNemar's Chi^2^ test was statistically significant (*p* < 0.05) in the majority of the considered inter-test agreement scenarios, indicating that the contingency tables for the two compared tests were not symmetrically distributed. Specifically, a bias was observed whereby the FPA was more likely to classify an animal as positive or doubtful for brucellosis, compared to the RBT.

## Discussion

This project sought to evaluate the efficiency and feasibility of using the FPA as a rapid on-spot test, and to compare its performance with the RBT, a more commonly used brucellosis test. Our experiences allowed us to identify a number of logistical and organizational issues when running the test in the field (slaughterhouse). Moreover, the moderate intra-test agreement and slight to poor inter-test agreement suggest that further adjustment in terms of temperature and ease of operation are needed before implementing the FPA in such settings.

As mentioned earlier, the rationale for this study was to determine whether the FPA could be used to screen and identify positive animals before slaughter, so that their organs could then be collected for further testing after. However, this proved to be challenging for a number of reasons. Firstly, poor restraining facilities made it difficult to collect blood from animals prior to slaughter, particularly from cattle. Therefore, blood was often collected during slaughter, and this subsequently reduced the time available for testing. Secondly, the lack of an animal identification system made it very hard to trace the sampled animals. This was further complicated by the fact that the skinning and dressing process was very quick, with the carcasses being released shortly after slaughter. Therefore, in many cases we returned to the slaughterhouse to find that the carcass and/or organs were no longer present. These experiences highlight how the success of such diagnostic and surveillance endeavors is dependent upon adequate logistic and sufficient human resources, both of which are often lacking in such settings (2, 6, 11, 20). Moreover, these findings were fundamental in informing the planning and design of subsequent epidemiological studies conducted in this study area.

One of the main advantages cited for the FPA is that, unlike other serological tests, it can be performed in the field, therefore expediting the testing process and reducing costs associated with transport of samples (12, 15). However, it is important to assess the suitability of a serological test locally, as contextual factors and climatic conditions may also influence the test's performance (20, 21). Therefore, with this project we sought to determine the feasibility of implementing the FPA in Abidjan, Côte d'Ivoire, a tropical country where brucellosis has been shown to be endemic at low levels, and no vaccination has been carried out in the last 20 years (22). We first performed the test in the laboratory using samples collected from animals in the livestock market. This allowed us to calibrate the device and finalize the testing protocol. All equipment was then carried to the slaughterhouse where a makeshift lab was set up in a room adjacent to the main slaughterhouse (Figure 2). This allowed us to process and test the samples within 20–30 min of collection.

The poor to moderate intra-test agreement between samples tested first in the slaughterhouse, and later in the laboratory, was unexpected since the test was carried out by the same person, following the same protocol, and using the same device and reagents. The only changes were in the location (slaughterhouse vs. laboratory) and time of testing (20–30 min vs. 6–8 h post-collection). A possible reason for the disagreement in the FPA results could be different working temperatures which can influence the molecular movement and, consequently, the fluorescence depolarization rate (5, 15, 23). In the laboratory we were able to maintain a constant ambient temperature of 22°C during execution of the FPA. However, this was not possible in the slaughterhouse makeshift laboratory, where it is likely that the room temperature reached 32°C (i.e., daily ambient temperature in Abdijan in April and May). To minimize this temperature effect, all reagents and samples were allowed to stand for a few minutes so as to ensure that they were all at the same temperature before testing. Moreover, control readings were taken at the beginning and end of each session, and after every 90–120 min if the session lasted longer than that, as per the manufacturers' instructions. Nonetheless, the higher temperatures in the slaughterhouse makeshift laboratory may have played a part in some of the different readings obtained.

We are unsure why the intra-test agreement improved when cattle samples were excluded, particularly since the FPA has primarily been described as a test for cattle (23, 24). The Kappa measurement is affected by the frequency of the finding under consideration, whereby lower Kappas are obtained when the frequency of the finding is either very high or very low (25). In this study, more small ruminants were classified as doubtful or positive for brucellosis with the FPA (33 and 26% of sheep and goats, respectively), compared to cattle (14%) (Table 1), and this may have led to the higher rate of Kappa agreement when cattle were excluded. However, work done in Kyrgyzstan also found that the FPA performed better in sheep and goats, compared to cattle (26, 27), which suggests that the type of conjugate used in the FPA, as well as unspecific cross-reactions, may also play a role in the different test performance.

All serum samples were retested with the RBT, a more widely used test which is simple to use, relatively cheap, and also provides rapid results within 10–20 min (6, 20). Our study found a poor to slight agreement between the FPA and RBT results, which was lower compared to that reported in other studies (20, 28). Bayasgalan (28) reports a Kappa of 0.56 between FPA and RBT carried out on cattle sera. However, this study was carried out in Mongolia, where both climatic conditions and management practices differ considerably from those in this study context. Muma et al. (20) also report a moderate Kappa agreement of 0.59 between FPA and RBT results for sera from traditionally reared cattle in Zambia, where conditions may be more comparable to those encountered in this study. However, both Muma et al. (20) and Bayasgalan (28) conducted the FPA under laboratory settings, which differs from this work where the goal was to assess the test's performance under field conditions. Nonetheless, the inter-agreement in this study improved only slightly when only FPA results obtained in the laboratory were considered (Kappa = 0.02–0.14). This highlights the need for proper standardization and validation of the FPA, even in laboratory conditions, particularly since both the ambient temperature where the test is conducted, and the temperature of the samples and reagents, can influence the test outcome.

When determining the inter-test agreement, the McNemar Chi^2^ test was often statistically significant, indicating asymmetry of the contingency tables. Specifically, the FPA was more likely to provide a positive result, compared to the RBT. This heightened sensitivity of the FPA may once again be due to the high temperatures, which affect the molecular rotation. Almost all Kappa agreements improved when the doubtful category was considered as negative, similar to findings reported by Bonfoh et al. (26). This further suggests that false positives by FPA, possibly due to the high ambient temperatures, could have led to the poor agreement between FPA and RBT.

In this study, the selection of animals was done purposively based on ease of restraining and the owners' willingness to participate. Moreover, the animal species were not equally represented, with fewer cattle sampled in the slaughterhouse, and none in the livestock markets. Therefore, the sample population was not randomly selected and not necessarily representative of the ruminant population in Abidjan. Nonetheless, since the aim of the study was to evaluate the feasibility and performance of the FPA, and no prevalence estimates were computed, this should not have compromised the study's internal validity. Similarly, as the main objective of this work was to evaluate the feasibility of using the FPA on the field prior to a larger epidemiological study planned for the same study area, and not to evaluate test characteristics or disease prevalence, no sample size calculations were calculated. Nonetheless, we believe that the number of samples obtained in this study was sufficient to provide us with conclusive results about the FPA's performance in this context.

Based on our experiences in this project, the FPA is not yet tailored to be used *in situ* in such low-resource settings and tropical conditions, and the effect temperature may have on the FPA readings needs to be further investigated. Specifically, the temperature of both the working environment, and all samples and reagents, should be monitored; and the same sera tested at different recorded temperatures should be evaluated. This would allow for the protocols to be adjusted and tailored to the different settings. In the meantime, the RBT continues to be a better screening alternative in such settings, as it is cheaper, less temperature-sensitive, and easier to process.

## Data Availability

The datasets generated for this study are available on request to the corresponding author.

## Ethics Statement

Approval was obtained from the National Ethics Committee of the Ministére de la Santé et de la Lutte contre le Sida of Côte d'Ivoire (N°71/MSLS/CNER-dkn) and the Direction Générale de Recherche Scientifique et de l'Innovation Technologique du Ministére de l'Enseignement Supérieur et de la Recherche Scientifique (N°089/MESRS/DGRSIT/KYS/tm). Authorization was also obtained from the district and the Directorate of Veterinary Services following a letter from the CSRS. Consent to sample was obtained from the animal owner, no animal owner information was collected, and all animal samples and results were coded and treated confidentially.

## Author Contributions

LF, BB, and ES conceived and designed the study. LF, ST, J-BA, and VK coordinated and conducted the field and laboratory activities. LF carried out the statistical analysis and prepared the first draft, while all co-authors revised and approved the final manuscript.

### Conflict of Interest Statement

The authors declare that the research was conducted in the absence of any commercial or financial relationships that could be construed as a potential conflict of interest.
